# The Effect of Exercise Training on Irisin Secretion in Patients with Type 2 Diabetes: A Systematic Review

**DOI:** 10.3390/jcm12010062

**Published:** 2022-12-21

**Authors:** Marco Vecchiato, Emanuele Zanardo, Francesca Battista, Giulia Quinto, Chiara Bergia, Stefano Palermi, Federica Duregon, Andrea Ermolao, Daniel Neunhaeuserer

**Affiliations:** 1 Sports and Exercise Medicine Division, Department of Medicine, University of Padova, Via Giustiniani 2, 35128 Padova, Italy; 2 Clinical Network of Sports and Exercise Medicine of the Veneto Region, 35131 Padova, Italy; 3 Public Health Department, University of Naples Federico II, 80131 Naples, Italy

**Keywords:** diabetes, FNDC5, physical activity, exercise prescription, high-intensity interval training

## Abstract

Introduction: Irisin is a myokine considered to be a potential mediator of exercise-induced energy metabolism and its secretion is known to promote the browning of beige fat cells in white adipose tissue. However, it is currently not known which exercise protocol is the best to enhance irisin concentration in patients with type 2 diabetes (T2D). Objective: The purpose of this study is to provide a review investigating the irisin response to different exercise training modalities and intensities in T2D. Methods: A systematic literature search was performed in May 2022. Results: After the selection process from 938 articles, six studies were included. Selected papers presented different exercise training interventions regarding intensity and modality. One study reported no significant differences in serum irisin levels after exercise training, whereas the other five showed a higher increase in serum irisin levels after exercise training with higher differences in irisin secretion after high-intensity training (HIT). No consideration was possible on exercise modalities. Conclusions: The impact of training intensity and modality was found to be partly discordant but data seem to suggest that HIT promotes greater irisin secretion. Despite the limited evidence, HIT, both in interval and continuous modalities, could be suggested as valid exercise training to increase circulating irisin in patients with T2D.

## 1. Introduction

**Figure 1:**
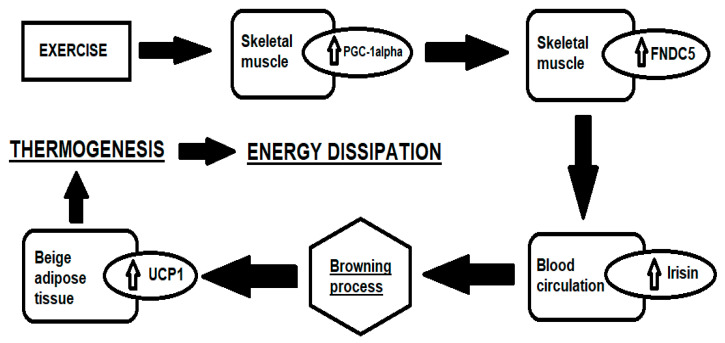
Simplified representation of the “browning” process [4].

Irisin is a myokine secreted into the bloodstream mainly by myocytes in response to muscle contraction and, in smaller quantities, by adipocytes. Irisin appears to contribute to the increase in energy expenditure via the conversion of white adipose tissue (WAT) into brown adipose tissue (BAT), achieved through the stimulation and up-regulation of the gene expression of uncoupling protein 1 (UCP1) [1]. From this conversion, an increase in thermogenesis is derived, thus promoting greater glucose tolerance, lower insulin resistance, and reduction in fat mass and body weight [2]. The irisin precursor has been identified in the fibronectin type 3 domain-containing protein 5 (FNDC5), a membrane protein which, when cleaved, generates irisin. Physical exercise induces an increase in peroxisome-proliferator-activated receptor gamma coactivator 1-alpha (PGC1-α), which stimulates the expression of the FNDC5 gene and thus the synthesis of irisin, causing an induction of the WAT “browning” process (Figure 1) [3].

Numerous studies have shown that irisin levels are lower in patients with type 2 diabetes (T2D) when compared with healthy subjects, even after adjusting for age, gender, and the subject’s body mass index (BMI) [5,6]. In fact, the FNDC5 synthesis is lower in T2D and, consequently, the secretion of irisin from adipose and muscle tissue will be reduced. The key factor underlying the lower secretion of irisin in the muscles is not clearly defined, but it is probably referable to the molecular cascade primed by chronic hyperglycemia and hyperlipidemia [3]. In support of this, it has been seen how irisin levels are negatively correlated with the levels of fasting blood glucose, 2hpG, HbA1c, and triglycerides [7]. The main cause of this pathological situation is due to the dysfunctional expression of PGC1-α in the myocytes of patients with T2D, which results in a reduced irisin secretion, playing an important role in so-called exercise resistance [2].

Different studies have investigated the effect of exercise training on healthy subjects describing a marked long-term reduction in T2D, obesity, and other chronic diseases [6,8]. An increase in the concentration of irisin, induced by exercise, is correlated, in all cases, with a higher energy expenditure and an improvement in glucose levels, obesity, and lipid profile [7,9]. This fact is mainly explained by the increase in the “browning” process, resulting from the increased stimulation by irisin [3].

Exercise modality and intensity are the main factors in stimulating specific metabolic pathways and muscle secretory mechanisms in response to exercise. Exercise training can include different exercise modalities such as aerobic training (AT) and resistance training (RT). AT is characterized by the execution of exercises with larger oxygen consumption and greater recruitment of red type I fibers. RT is characterized by the execution of exercises against an external force with predominant recruitment of white type II fibers. High-intensity interval training (HIIT) is a new form of high intensity training (HIT) modality, performed with short maximal or submaximal efforts and light intermittent recovery periods, which is currently receiving increasing attention regarding its metabolic and cardiovascular effects [10,11].

Despite the demonstrated effects of exercise training on irisin production in healthy subjects, it remains unclear how this works in patients with T2D. This study aims to highlight which exercise modality between AT and RT and which exercise intensity between HIT (both continuous and interval) and moderate intensity training (MIT) is better to increase irisin levels compared with baseline in adult patients with T2D.

## 2. Materials and Methods

### 2.1. Focused Question

Which exercise modality (AT vs. RT) and intensity (HIT vs. MIT) is most effective on irisin secretion in community-dwelling adult patients with T2D?

### 2.2. Databases

Four electronic databases (PubMed, Cochrane Library, Embase, and SportDiscus) were searched for original articles published up to May 2022 using a specific search strategy. The search term combination was built using the Boolean operators “AND” and “OR”, in order to integrate and reach the maximum number of related studies; the key words used for the search are reported in Appendix A. Reference lists from the resulting articles were also screened to identify additional articles. The PRISMA (Preferred Reporting Items for a Systematic Review and Meta-Analysis) guideline for improving the strategy of paper screening was followed [12].

### 2.3. Study Selection

This systematic review included only studies that reported the effect of exercise training on irisin secretion. The inclusion criteria comprised randomized clinical trials (RCT), and double-blind and placebo-controlled studies that reported the use of exercise training protocols in patients with T2D in which the pre- and post-intervention blood irisin concentration was assessed. Non-randomized treatment assignment was considered as an exclusion criterion. Only full texts were included. The PICO (population, intervention, comparison, and outcomes) format was followed for this review (Table 1). In vitro studies, studies with animals, reviews, studies not published in English, case reports, and editorials were excluded. Studies on subjects with glucose intolerance or metabolic syndrome in which T2D was not among the inclusion criteria were not selected. The minimum length of the intervention period was considered as 4 weeks. The presence of co-interventions in addition to exercise training was not considered as an exclusion criterion.

### 2.4. Data Extraction

The search period for this review included the past ten years (May 2012 to May 2022). Data from figures were extracted using an online tool (WebPlotDigitizer; https://automeris.io/WebPlotDigitizer/, accessed on 17 November 2022).

### 2.5. Quality Assessment

The risk of bias of the included studies was assessed using the revised Cochrane risk-of-bias tool for randomized trials (RoB 2.0) [13]. During the review process, the authors applied the tool to each included study. An overall summary risk-of-bias judgment (low; some concerns; high) was derived, whereby the overall RoB score for each study was determined by the highest RoB level in any of the domains that were assessed. Quality assessment was conducted independently by two reviewers (EZ and MV) using this standardized tool. Any disagreement between the reviewers was resolved through discussion.

## 3. Results

**Figure 2:**
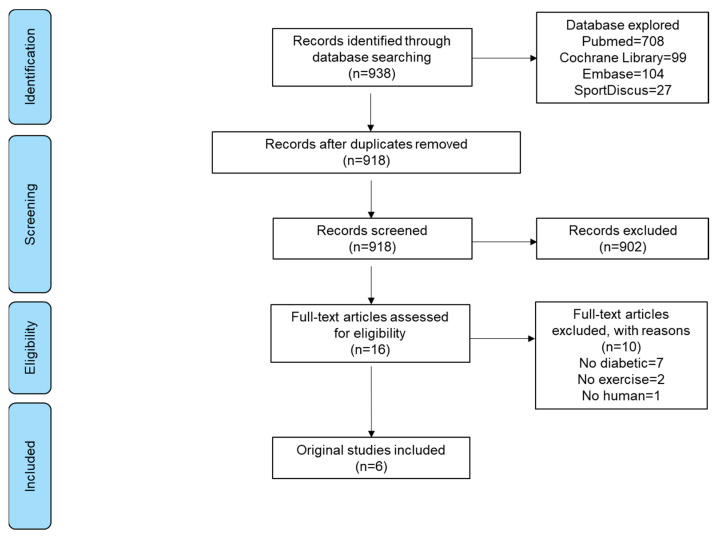
Preferred Reporting Items for Systematic Reviews and Meta-Analysis (PRISMA) flow diagram.

The flow diagram of this systematic review is presented in Figure 2. The comprehensive search yielded 938 articles; 20 articles were removed because they were duplicates. Of the remaining 918, 902 were eliminated after reading the titles and abstracts, according to the eligibility criteria. The full text was retrieved from 16 articles, of which six satisfied the inclusion criteria.

The selected studies are shown in Table 2. From the six articles selected, a total of 219 participants were included [14,15,16,17,18,19]. Five studies presented a control group without exercise-training intervention [15,16,17,18,19]. The median (range) total sample size of the included studies was 38 (14–60) subjects. It was not possible to calculate the mean age because one study did not report the required data. However, the median age should rest between 50 and 60 years. Of the participants, 118 were women (54%) and 101 were men (46%); two studies included only women [15,16] and one only men [19]. In the studies including both genders the mean percentage of females was 46% [14].

All participants were patients with diagnosed T2D. One study also presented an additional control group not affected by T2D which has not been considered for the purposes of this review. Four studies reported how many people were on anti-hypoglycemic therapy (87% of the total) [14,16,17,19]. Three studies reported insulin treatment among the exclusion criteria [14,17,19]. One study indicated having a history of diabetes with insulin injection for more than 5 years as an inclusion criterion [15]. Just two studies reported the duration of T2D (mean duration 6 years) [14,17]. Five studies excluded patients with a history of chronic diseases other than T2D (cardiovascular disease including untreated severe hypertension or arrhythmias, thyroid disorders, cancer, or kidney or liver disease) and smokers [14,16,17,18,19]. Three studies recruited individuals who were overweight or obese (BMI > 25 kg/m^2^) [16,17,19]. One study did not report the BMI of participants [15]. One study did not specify exclusion criteria [15].

The duration of the exercise training intervention ranged from 4 to 16 weeks, with a median duration of 11 weeks and a median of 3 (range 3–5) sessions/week. One study performed only AT with different intensities [14], whereas combined AT and RT were performed in the remaining studies [15,16,17,18,19] and HIIT was used in three studies [14,16,19]. Exercise duration was prescribed in minutes and the exercise intensity defined as percentage of maximal heart rate (HRmax) or maximal aerobic capacity (VO_2_max) for AT; a percentage of the 1 repetition maximum (1-RM) or different elasticity resistance bands were used for RT.

The mean (range) exercise duration was 131 (30–375) min per week at 69% (60–75%) of HRmax for AT. Two studies prescribed AT referring to 60% of VO_2_max for moderate intensity and 85% for high intensity [14,18]. One study prescribed exercise intensity based only on maximal oxygen uptake percentage obtained from cardiopulmonary exercise testing [17]. RT was prescribed with a median (range) of 2 (1–3) sets of 15 (10–20) repetitions each. Two studies included five and six exercises on weightlifting machines [16,19] and one study included elastic bands [15]. Exercises with machines were performed at 60% and 65% of 1-RM, whereas the resistance of the elastic bands was different between moderate- and high-intensity training groups (starting from 100% and 125%, respectively). One study designed the training protocol following the recommendations of the American College of Sports Medicine and American Diabetes Association for subjects with T2D [17].

HIIT was prescribed with a median (range) of 5 (4–10) bouts of 70 (30–120) s at 92 (from 85 to maximal) % VO_2_max or 92 (from 85 to maximum) % HRmax with a median (range) intermittent recovery duration of 65 (60–90) s at 60% VO_2_max, 65% HRmax, or 50 Watt. HIIT warm-up and recovery phases were conducted on 60% of VO_2_max.

Exercise sessions were fully supervised in four studies [14,16,17,19]. Information about supervision was not reported in the remaining studies [15,18]. Control groups were characterized by no exercise prescription, usual care, or diabetes recommendations for self-management; patients were asked to maintain baseline activity levels during the study period.

### 3.1. Irisin

Baseline blood samples were collected about 48h (24–120 h) before the first training session. Four studies specified that blood samples were obtained after a 12 h overnight fast and before medication intake in the early morning [16,19]. Post-study blood samples were collected after a median (range) of 48 h (24–120 h) from the end of the training sessions. One study did not report the timing of the two sample collections [15]. In all studies, irisin was measured from venous blood using a commercially available enzyme-linked ELISA kit. Samples were processed immediately and kept at −80 °C until time of analysis.

Five studies reported blood irisin concentration at baseline with a median (range) value of about 438 (8.77–1400) ng/mL. Post-exercise-training blood irisin concentrations were 464 (8.85–1575) ng/mL [14,16,17,19]. One study reported low irisin blood levels (18 pg/mL and 25 pg/mL before and after intervention, respectively) [18]. In the single study not reporting blood irisin values of the subdivision groups, blood irisin concentration at baseline was between 3.47 and 4.50 µg/mL, whereas the post-study blood irisin concentration was between 3690 and 21,030 ng/mL [15].

One study, despite the increase observed in the two intervention groups, showed no significant effect for serum irisin [16]. Four studies reported a significant increase in resting plasma irisin concentration for the exercise intervention group compared with controls [15,17,18,19]. Exercise intervention groups in these studies presented an average variation (Δ%) in blood irisin level of +39% compared with the −5% of control groups [15,17,18,19]. A comparison of the Δ% between the AT and RT groups was not possible as no study had groups that only performed strength exercises. In the single study not reporting the subdivision groups, Δ% in serum irisin concentration within groups was +88% and +368% for the moderate- and high-intensity groups, respectively, compared with the +6% of the control group [16]. In the four studies with a high-intensity exercise group, the HIT intervention showed an average Δ% in blood irisin level of +114% compared with the average +49% of the MIT intervention [14,15,16,19]. In the three exercise groups with an interval protocol, HIIT intervention showed an average Δ% in blood irisin level of +18% comparable to the +16% of MIT intervention groups [14,16,19].

### 3.2. Quality Assessment

**Figure 3:**
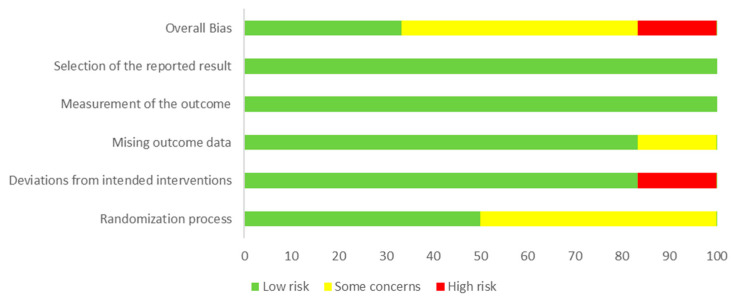
Risk-of-bias graph: judgments about each item related to the risk of bias and presented as percentages across all included studies.

Two of the studies included were judged to have a low overall RoB score, three with a RoB score of “some concerns”, and one with a high RoB score, deriving from deviations from intended study interventions, missing outcome data, altered measurement of the outcome, and selection of the reported results. The evaluation of the methodological quality of the studies included is shown in Figure 3.

## 4. Discussion

This systematic review provides a comprehensive analysis on the effect of exercise training interventions on irisin secretion in people with T2D. To date, the studies specifically investigating the effect of exercise on irisin secretion in T2D are few and the overall evidence can be summarized as follows:

1. Exercise intervention increases irisin secretion in individuals with T2D.
2. There is not yet sufficient evidence in the literature to determine which exercise training modality results in a greater secretion of irisin in this population.
3. Data on the training intensity are partly discordant but they suggest that high intensity exercise facilitates greater secretion of irisin.
4. Several factors, such as timing of the samplings, disease history, and the body composition of the patients, represent a large source of variability among the studies reported.

### 4.1. Influence of Training Modality and Intensity on Irisin Level in T2D

Training modality is one of the main factors stimulating specific metabolic pathways and muscle secretory mechanisms. It has been shown that mitochondrial biogenesis is increased following an AT intervention as a direct consequence of the increase in PGC1-α levels, whereas RT does not seem to induce a PGC1-α upregulation in equal measure. Blood irisin levels recorded following an AT session, for both endurance and HIIT, were found to be higher when compared with those recorded after an RT session [20]. On the other hand, some studies showed how only RT positively and significantly influences the irisin concentration [7,21]. A recent systematic review and meta-analysis analyzing the effect of exercise interventions on irisin level showed how isolated RT and combined AT and RT appear to be the optimal stimulus to increase irisin levels [22]. In all studies included in this systematic review, RT was administered together with AT, thus it was not possible to discriminate between the effects of the two exercise modalities on blood irisin levels. One study recruited two groups of patients who underwent AT-RT and RT-AT sequences, showing no differences between them in irisin increase compared with baseline but both showing differences compared with the control group [19]. It appears that the order of training modality makes no difference in the response of the irisin [23]. Two of the reviewed studies presented a comparison between interval and continuous training with one of the two showing a greater effect of interval training on circulating irisin levels [14,16]. One study compared the same exercise protocol at different intensities (moderate- vs. high-intensity combined AT and RT) demonstrating higher irisin secretion after the high-intensity training program [15]. An RCT performed by Huh and colleagues showed that all the investigated exercise modalities (AT, RT, and HIIT) positively affected the acute post-exercise irisin levels; in particular, RT appeared to be the most effective, both in healthy subjects and in those affected by metabolic syndrome [24]. Different results derived from Fox and colleagues who found no significant correlation between the post-exercise irisin level and the training method [25]. In support of this, a study by Pekkala and colleagues compared low-intensity AT, heavy-intensity AT, and long-term endurance training with and without RT, describing that the increase in irisin concentration following the various training sessions was not significantly different [26].

The present systematic review suggests that HIT, not necessarily administered with an interval protocol, may be more effective in increasing irisin in patients with T2D. Previous studies compared the effects on irisin concentrations resulting from HIT and MIT. Among these studies, Fatouros and colleagues showed that irisin concentration increased acutely in all exercise groups with a dose-dependent increase in irisin concentration with the intensity of training [20]. Intensity and duration of exercise seem to be positively correlated with the increase in serum irisin concentration, regardless of energy consumption [27]. This must be taken into consideration in identifying the best type of training to be adopted in order to obtain the maximum secretion of irisin in the population with T2D. Therefore, it is plausible that in patients with T2D, higher intensity or longer duration of AT or RT are required. However, current evidence is limited and yet not clearly indicating which type of workouts could better affect irisin levels in this specific population [28].

### 4.2. Exercise Training Effect on Irisin Secretion in T2D Patients: Sources of Variability

Several factors can help to explain the large variability found in the selected studies concerning the values of circulating irisin after a period of exercise training. First of all, it must be pointed out that the timing of the sampling in relation to the last training session appears to be a determining factor. Indeed, several studies investigating the acute effect of exercise on irisin secretion in healthy subjects have shown a significant increase [29,30,31]. In particular, a rise of 20% was noted in irisin levels following moderate-intensity AT and of 18% following anaerobic exercise characterized by a “sprint-running session” [32]. Furthermore, most of the studies agree in limiting the effect of physical exercise on irisin levels only for the acute post-exercise phase; in the chronic phase, the effects of physical exercise on the irisin concentration seem to decrease up to complete cessation [33]. However, some studies also documented a chronic rise in post-exercise irisin levels in the long term [3]. Conversely, the studies selected in the present systematic review showed heterogeneous effects on long-term circulating irisin in patients with T2D. Discordant results have also been obtained regarding the increase in circulating irisin levels following chronic physical exercise in the healthy population [29]. To this should be added the great variability described in the literature regarding the concentration of circulating irisin in healthy humans, as different studies reported a wide-ranging variability using different measurement kits [7,34].

Moreover, the subject’s profile and the healthy status may influence the irisin response to exercise. It has been previously described that the increase in the post-exercise expression of FNDC5 is higher (>30%) in active subjects compared with their sedentary counterparts or with patients affected by chronic diseases [35]. It can be deduced that patients with T2D, following physical exercise, can obtain a lower increase in irisin secretion than healthy subjects but it is probably still relevant for the improvement of their health status [36]. On the other hand, other studies have shown how the beneficial effect of physical exercise on irisin levels is limited only to populations with certain characteristics, including the presence of metabolic diseases [4]. An RCT by Micielska and colleagues conducted on sedentary adult women with diminished insulin sensitivity showed an increase in the irisin values before and after five weeks of circuit HIIT only in the group containing old participants [37]. Conversely, in young patients in the training group, irisin levels were even found to be reduced after exercise. Therefore, age is probably a determining factor for the irisin response to exercise. The selected studies showed a large variability in terms of age groups and inclusion criteria. Indeed, the studies also differed greatly regarding patients’ comorbidities being considered as exclusion criteria, but most importantly, insulin-dependent diabetes was considered as one of the inclusion criteria in some studies [15,16], and as an exclusion criterion in others [14,17,19]. Insulin therapy is likely to represent a more advanced stage of the disease that may present greater severity and related complications, a source of further variability. In fact, the duration of T2D and its complications/comorbidities should also be considered. It is reasonable that individuals with long-standing and more severe diabetes, in terms of both glycemic control and complications, have lower irisin production in response to exercise [32,38]. Only two studies reported disease duration and in one of them patients did not show an increase in irisin secretion after an exercise training period [14,17]. Moreover, reduced circulating irisin levels have been described in patients affected by chronic kidney disease and non-alcoholic fatty liver disease [39,40]. None of the selected studies provided data on the kidney or liver function of the participants, limiting any further considerations.

A further source of variability is related to the duration of exercise training. Two studies on the same sample of overweight women with metabolic syndrome showed that an increase in blood irisin levels was present at 12 weeks but not at 8 weeks after exercise training [41,42]. Paradoxically, in our review the study with the shortest intervention duration was the one with the largest percentage increase [15].

Moreover, BMI and body composition may also influence the irisin level; therefore, patients would need to be better characterized and standardized in order to identify the real effect of exercise in determining circulating irisin, especially in training intervention studies, which in turn can determine changes in body weight and body composition [43,44]. Baseline circulating irisin levels are significantly and positively correlated with BMI, metabolic syndrome components, and insulin resistance; this supports the concept that higher irisin levels are associated with increase in body weight, which is also associated with an increase in WAT [4,32]. Furthermore, the elevation of irisin in overweight subjects can be explained by the mechanism of “irisin resistance”, in which the organism produces a compensatory excess of irisin to satisfy metabolic demands, similar to the concept of insulin resistance [45,46]. On the other hand, several characteristics of T2D, such as hyperglycemia, hypertriglyceridemia, visceral adiposity, and extramyocellular lipid deposition, were negatively associated with adipose tissue FNDC5 mRNA and circulating irisin [47]. The role of irisin in glycemic control is still unclear but it may be reasonable to hypothesize that lower levels of circulating irisin in patients with T2D may be due to impaired PGC-1α expression and functions in their muscle tissue [2]. It is possible that even within the adipose tissue of overweight patients with T2D, the irisin secretion is decreased due to the inflammatory processes typical of obesity [48].

HIIT training seems superior for improving cardiopulmonary fitness and to reduce the percentage of body fat in adults with obesity compared with traditional exercise [49,50]. This could be particularly useful for patients with obesity and T2D as they show poor metabolic flexibility or specific responses to exercise depending on possible comorbidities [51,52].

Furthermore, it must be emphasized that the quality of the studies was limited and the study designs very different. This is a further element of bias and may explain the variability in outcomes. Clinical trials using exercise as an intervention are already prone to great variability for all the reasons intrinsically linked to the administration of exercise; adding molecular blood analyses with different protocols and sampling times will only increase this already wide variability.

Further studies are needed to better understand the effect of the various training modalities and intensities on irisin secretion and to establish the molecular effects of exercise training on patients with T2D.

## 5. Conclusions

All the different types of exercise seem to increase circulating irisin levels in people with T2D. There is not yet sufficient evidence to clearly determine the best exercise modality to increase irisin secretion in patients with T2D. Moreover, it seems that HIT exercise is associated with higher levels of irisin secretion. Therefore, HIT, both in interval and continuous modalities, could be proposed as potential effective training protocols in T2D in order to increase irisin levels. Overall, physical exercise, combined with drug therapy and a healthy diet, could help to enhance irisin secretion and thus to increase energy expenditure, reduce body weight, and improve body composition in patients with T2D.

## Figures and Tables

**Table 1:** PICO components. T2D: type 2 diabetes; AT: aerobic training; RT: resistance training; HIT: high intensity training; MIT: moderate intensity training; PICO: population, intervention, comparison, and outcomes.

Population	Intervention	Comparison	Outcome
Community-dwelling adult patients with T2D	Exercise training	1. Exercise training vs. controls 2. AT vs. RT 3. HIT vs. MIT	Irisin secretion compared with baseline

**Table 2:** General overview of included studies. Abbreviations: 1-RM: one repetition maximum; AT: aerobic training; C(M/H)T: continuous (moderate/high-intensity) training; HIIT: high-intensity interval training; HRmax: maximal heart rate; RT: resistance training; T2D: type 2 diabetes; VO_2_max: maximal aerobic capacity; Δ irisin concentration: variation between pre- and post-intervention in blood irisin concentration (expressed in %).

Author and Year	Population	Age and Gender	T2D Duration (Years)	Number of Participants (Group Distribution)	Study Intervention	Type of Study	Intensity	Supervised	Training Duration (weeks)	Training Volume (min/week)	Antidiabetic Drugs Treatment	Blood Sample Collection	Δ Irisin Concentration	Findings
Banitalebi et al., 2019 [16]	T2D; Overweight or obese; sedentary.	30–65 y; only women	nr	n = 42 (HIIT = 14; RT + AT = 14; controls = 14)	HIIT vs. RT + AT vs. controls	RCT	RT + AT: 1-RM + 60%HRmax; HIIT: 100% of perceived exertion	y	10	150	Oral (n = 27) +Insulin (n = 20) +Combined (n = 5)	48 h after intervention period	HIIT: +13.1% RT + AT: +23.6% Controls: +6.5%	No significant differences in serum irisin between groups
Dünnwald et al., 2019 [14]	T2D	50–65 y; both genders	HIIT: 10.7 ± 4.6; CMT: 6.9 ± 4.3	n = 14 (HIIT = 8 vs. CMT = 6)	HIIT vs. CMT	nRCT	HIIT: 90–95% HRmax (85% VO_2_max); CMT: 70%HRmax (60% VO_2_max)	y	4	126	Oral (n = 11)	1 day after intervention period	HIIT: +7% CMT: −1.7%	HIIT but not CMT increases the serum irisin concentration
Enteshary et al., 2019 [15]	T2D	35–45 y; only women	nr	n = 26 divided in 3 groups (controls vs. CMT vs. CHT)	CMT vs. CHT vs. controls	RCT	CMT: 62%HRmax; CHT: 80%HRmax	nr	8	150 CMT-375 CHT	Insulin (n not reported)	nr	CMT: +88% CHT: +367% Controls: +6%	CMT and CHT both increase serum irisin. CHT seems to be more effective.
Motahari Rad et al., 2020 [19]	T2D	40–50 y; only men	nr	n = 43 (RT + AT = 15; AT + RT = 15; controls = 13)	RT + AT vs. AT + RT vs. controls	RCT	RT: from 40% to 80% of 1-RM; AT: 75–95%HRmax	y	12	30 (3 times per week of 10 × 1 min HIIT-rest)	Oral (n = 43)	48h after intervention period	RT + AT: +18.7% AT + RT: +27.2% Controls: −4.5%	Both RT + AT and AT + RT groups increase irisin compared with controls
Bonfante et al., 2022 [17]	T2D; Overweight; sedentary	40–60 y; both genders	RT + AT: 5.5 ± 2.62; controls: 4.94 ± 3.05	n = 34 (RT + AT = 17; controls = 17)	RT + AT vs. controls	RCT	RT: 1–3 sets of submaximal exercise, AT: 35min at 50–70% of VO_2_max)	nr	16	RT: 120; AT: 105	Oral (n = 60)	nr	RT + AT: +27.5% Controls: −7.5%	Combined training increases serum irisin
Yang et al., 2020 [18]	T2D	Exercise group: 47.7 ± 7.4 y; Controls: 45.2 ± 8.8 y; both genders	nr	n = 60 (AT + RT = 30; controls = 30)	AT (+non enforced RT) vs. controls	RCT	Moderate AT: 60% VO_2_max; RT: nr	y	12	150	Oral (n = ?)	nr	AT(+RT): +97% Controls: −8%	Exercise group results in an improvement of the serum irisin compared with controls

## Data Availability

Not applicable.

## Appendix A

((diabetes type 2 OR glucose intolerance OR metabolic syndrome OR pancreatic cell OR insulin resistance)) AND ((irisin OR FNDC5 OR PGC1-α OR browning)) AND ((exercise OR training OR HIIT OR physical activity OR sport)).

## References

[1] Boström P., Wu J., Jedrychowski M.P., Korde A., Ye L., Lo J.C., Rasbach K.A., Boström E.A., Choi J.H., Long J.Z. (2012). A PGC1-α-dependent myokine that drives brown-fat-like development of white fat and thermogenesis. Nature.

[2] Shoukry A., Shalaby S.M., El-Arabi Bdeer S., Mahmoud A.A., Mousa M.M., Khalifa A. (2016). Circulating serum irisin levels in obesity and type 2 diabetes mellitus. IUBMB Life.

[3] Perakakis N., Triantafyllou G.A., Fernández-Real J.M., Huh J.Y., Park K.H., Seufert J., Mantzoros C.S. (2017). Physiology and role of irisin in glucose homeostasis. Nat. Rev. Endocrinol..

[4] Hofmann T., Elbelt U., Stengel A. (2014). Irisin as a muscle-derived hormone stimulating thermogenesis—A critical update. Peptides.

[5] Du X.L., Jiang W.X., Lv Z.T. (2016). Lower Circulating Irisin Level in Patients with Diabetes Mellitus: A Systematic Review and Meta-Analysis. Horm. Metab. Res..

[6] Liu J.-J., Wong M.D., Toy W.C., Tan C.S., Liu S., Ng X.W., Tavintharan S., Sum C.F., Lim S.C. (2013). Lower circulating irisin is associated with type 2 diabetes mellitus. J. Diabetes Complicat..

[7] Choi Y.-K., Kim M.-K., Bae K.H., Seo H.-A., Jeong J.-Y., Lee W.-K., Kim J.-G., Lee I.-K., Park K.-G. (2013). Serum irisin levels in new-onset type 2 diabetes. Diabetes Res. Clin. Pract..

[8] Pedersen B.K., Saltin B. (2015). Exercise as medicine—Evidence for prescribing exercise as therapy in 26 different chronic diseases. Scand. J. Med. Sci. Sports.

[9] Hwang Y.C., Jeon W.S., Park C.Y., Youn B.S. (2016). The ratio of skeletal muscle mass to visceral fat area is a main determinant linking circulating irisin to metabolic phenotype. Cardiovasc. Diabetol..

[10] Ramos J.S., Dalleck L.C., Tjonna A.E., Beetham K.S., Coombes J.S. (2015). The impact of high-intensity interval training versus moderate-intensity continuous training on vascular function: A systematic review and meta-analysis. Sports Med..

[11] Way K.L., Sabag A., Sultana R.N., Baker M.K., Keating S.E., Lanting S., Gerofi J., Chuter V.H., Caterson I.D., Twigg S.M. (2020). The effect of low-volume high-intensity interval training on cardiovascular health outcomes in type 2 diabetes: A randomised controlled trial. Int. J. Cardiol..

[12] Page M.J., McKenzie J.E., Bossuyt P.M., Boutron I., Hoffmann T.C., Mulrow C.D., Shamseer L., Tetzlaff J.M., Akl E.A., Brennan S.E. (2021). The PRISMA 2020 statement: An updated guideline for reporting systematic reviews. BMJ.

[13] Higgins J.P.T., Savović J., Page M.J., Elbers R.G., Sterne J.A.C. (2019). Assessing risk of bias in a randomized trial. Cochrane Handbook for Systematic Reviews of Interventions.

[14] Dünnwald T., Melmer A., Gatterer H., Salzmann K., Ebenbichler C., Burtscher M., Schobersberger W., Grander W. (2019). Supervised Short-term High-intensity Training on Plasma Irisin Concentrations in Type 2 Diabetic Patients. Int. J. Sports Med..

[15] Enteshary M., Esfarjani F., Reisi J. (2019). Comparison of the Effects of Two Different Intensities of Combined Training on Irisin, Betatrophin, and Insulin Levels in Women with Type 2 Diabetes. Asian J. Sports Med..

[16] Banitalebi E., Kazemi A.R., Faramarzi M., Nasiri S., Haghighi M.M. (2019). Effects of sprint interval or combined aerobic and resistance training on myokines in overweight women with type 2 diabetes: A randomized controlled trial. Life Sci..

[17] Bonfante I.L.P., Monfort-Pires M., Duft R.G., Mateus K.C.D.S., Júnior J.C.D.L., Trombeta J.C.D.S., Finardi E.A.R., Brunelli D.T., Morari J., de Lima J.A.B. (2022). Combined training increases thermogenic fat activity in patients with overweight and type 2 diabetes. Int. J. Obes..

[18] Yang D., Li Y., Fan X., Liang H., Han R. (2020). The impact of exercise on serum irisin, osteocalcin, and adiponectin levels and on glycolipid metabolism in patients with type 2 diabetes. Int. J. Clin. Exp. Med..

[19] Motahari Rad M., Bijeh N., Attarzadeh Hosseini S.R., Raouf Saeb A. (2020). The effect of two concurrent exercise modalities on serum concentrations of FGF21, irisin, follistatin, and myostatin in men with type 2 diabetes mellitus. Arch. Physiol. Biochem..

[20] Fatouros I.G. (2018). Is irisin the new player in exercise-induced adaptations or not? A 2017 update. Clin. Chem. Lab. Med..

[21] Cosio P.L., Crespo-Posadas M., Velarde-Sotres Á., Pelaez M. (2021). Effect of Chronic Resistance Training on Circulating Irisin: Systematic Review and Meta-Analysis of Randomized Controlled Trials. Int. J. Environ. Res. Public Health.

[22] Rahimi G.R.M., Hejazi K., Hofmeister M. (2022). The effect of exercise interventions on Irisin level: A systematic review and meta-analysis of randomized controlled trials. EXCLI J..

[23] Kim H.J., Lee H.J., So B., Son J.S., Yoon D., Song W. (2016). Effect of aerobic training and resistance training on circulating irisin level and their association with change of body composition in overweight/obese adults: A pilot study. Physiol. Res..

[24] Huh J.Y., Siopi A., Mougios V., Park K.H., Mantzoros C.S. (2015). Irisin in response to exercise in humans with and without metabolic syndrome. J. Clin. Endocrinol. Metab..

[25] Fox J., Rioux B.V., Goulet E.D.B., Johanssen N.M., Swift D.L., Bouchard D.R., Loewen H., Sénéchal M. (2018). Effect of an acute exercise bout on immediate post-exercise irisin concentration in adults: A meta-analysis. Scand. J. Med. Sci. Sports.

[26] Pekkala S., Wiklund P.K., Hulmi J.J., Ahtiainen J.P., Horttanainen M., Pöllänen E., Mäkelä K.A., Kainulainen H., Häkkinen K., Nyman K. (2013). Are skeletal muscle FNDC5 gene expression and irisin release regulated by exercise and related to health?. J. Physiol..

[27] Gamas L., Matafome P., Seicą R. (2015). Irisin and Myonectin Regulation in the Insulin Resistant Muscle: Implications to Adipose Tissue: Muscle Crosstalk. J. Diabetes Res..

[28] Parada-Sánchez S.G., Macias-Cervantes M.H., Pérezvázquez V., Vargas-Ortiz K. (2022). The Effects of Different Types of Exercise on Circulating Irisin Levels in Healthy Individuals and in People with Overweight, Metabolic Syndrome and Type 2 Diabetes. Physiol. Res..

[29] Qiu S., Bosnyák E., Treff G., Steinacker J.M., Nieß A.M., Krüger K., Mooren F.C., Zügel M., Schumann U. (2018). Acute exercise-induced irisin release in healthy adults: Associations with training status and exercise mode. Eur. J. Sport Sci..

[30] Norheim F., Langleite T.M., Hjorth M., Holen T., Kielland A., Stadheim H.K., Gulseth H.L., Birkeland K.I., Jensen J., Drevon C.A. (2014). The effects of acute and chronic exercise on PGC-1α, irisin and browning of subcutaneous adipose tissue in humans. FEBS J..

[31] Kraemer R.R., Shockett P., Webb N.D., Shah U., Castracane V.D. (2014). A transient elevated irisin blood concentration in response to prolonged, moderate aerobic exercise in young men and women. Horm. Metab. Res..

[32] Huh J.Y., Panagiotou G., Mougios V., Brinkoetter M., Vamvini M.T., Schneider B.E., Mantzoros C.S. (2012). FNDC5 and irisin in humans: I. Predictors of circulating concentrations in serum and plasma and II. mRNA expression and circulating concentrations in response to weight loss and exercise. Metabolism.

[33] Qiu S., Cai X., Sun Z., Schumann U., Zügel M., Steinacker J.M. (2015). Chronic Exercise Training and Circulating Irisin in Adults: A Meta-Analysis. Sports Med..

[34] Moreno-Navarrete J.M., Ortega F.J., Serrano M., Guerra E., Pardo G., Tinahones F., Ricart W., Fernández-Real J.M. (2013). Irisin is expressed and produced by human muscle and adipose tissue in association with obesity and insulin resistance. J. Clin. Endocrinol. Metab..

[35] Timmons J.A., Baar K., Davidsen P.K., Atherton P.J. (2012). Is irisin a human exercise gene?. Nature.

[36] Vecchiato M., Quinto G., Palermi S., Foccardi G., Mazzucato B., Battista F., Duregon F., Michieletto F., Neunhaeuserer D., Ermolao A. (2022). Are Gyms a Feasible Setting for Exercise Training Interventions in Patients with Cardiovascular Risk Factors? An Italian 10-Years Cross-Sectional Survey Comparison. Int. J. Environ. Res. Public Health.

[37] Micielska K., Kortas J.A., Gmiat A., Jaworska J., Kozlowska M., Lysak-Radomska A., Rodziewicz-Flis E., Zychowska M., Ziemann E. (2021). Habitually inactive physically—A proposed procedure of counteracting cognitive decline in women with diminished insulin sensitivity through a high-intensity circuit training program. Physiol. Behav..

[38] Moreno M., Moreno-Navarrete J.M., Serrano M., Ortega F., Delgado E., Sanchez-Ragnarsson C., Valdés S., Botas P., Ricart W., Fernández-Real J.M. (2015). Circulating irisin levels are positively associated with metabolic risk factors in sedentary subjects. PLoS ONE.

[39] Wen M.S., Wang C.Y., Lin S.L., Hung K.C. (2013). Decrease in Irisin in Patients with Chronic Kidney Disease. PLoS ONE.

[40] Zhang H.-J., Zhang X.-F., Ma Z.-M., Pan L.-L., Chen Z., Han H.-W., Han C.-K., Zhuang X.-J., Lu Y., Li X.-J. (2013). Irisin is inversely associated with intrahepatic triglyceride contents in obese adults. J. Hepatol..

[41] Dianatinasab A., Koroni R., Bahramian M., Bagheri-Hosseinabadi Z., Vaismoradi M., Fararouei M., Amanat S. (2020). The effects of aerobic, resistance, and combined exercises on the plasma irisin levels, HOMA-IR, and lipid profiles in women with metabolic syndrome: A randomized controlled trial. J. Exerc. Sci. Fit..

[42] Amanat S., Sinaei E., Panji M., MohammadporHodki R., Bagheri-Hosseinabadi Z., Asadimehr H., Fararouei M., Dianatinasab A. (2020). A Randomized Controlled Trial on the Effects of 12 Weeks of Aerobic, Resistance, and Combined Exercises Training on the Serum Levels of Nesfatin-1, Irisin-1 and HOMA-IR. Front. Physiol..

[43] Stengel A., Hofmann T., Goebel-Stengel M., Elbelt U., Kobelt P., Klapp B.F. (2013). Circulating levels of irisin in patients with anorexia nervosa and different stages of obesity–Correlation with body mass index. Peptides.

[44] Liu J.-J., Liu S., Wong M.D., Tan C.S., Tavintharan S., Sum C.F., Lim S.C. (2014). Relationship between circulating irisin, renal function and body composition in type 2 diabetes. J. Diabetes Complicat..

[45] Park K.H., Zaichenko L., Brinkoetter M., Thakkar B., Sahin-Efe A., Joung K.E., Tsoukas M., Geladari E.V., Huh J.Y., Dincer F. (2013). Circulating Irisin in Relation to Insulin Resistance and the Metabolic Syndrome Division of Endocrinology, Diabetes, and Metabolism (K). J. Clin. Endocrinol. Metab..

[46] Polyzos S.A., Kountouras J., Shields K., Mantzoros C.S. (2013). Irisin: A renaissance in metabolism?. Metabolism.

[47] Kurdiova T., Balaz M., Vician M., Maderova D., Vlcek M., Valkovic L., Srbecky M., Imrich R., Kyselovicova O., Belan V. (2014). Effects of obesity, diabetes and exercise on Fndc5 gene expression and irisin release in human skeletal muscle and adipose tissue: In vivo and in vitro studies. J. Physiol..

[48] Al-Daghri N.M., Alokail M.S., Rahman S., Amer O.E., Al-Attas O.S., Alfawaz H., Tripathi G., Sabico S., Chrousos G.P., McTernan P.G. (2015). Habitual physical activity is associated with circulating irisin in healthy controls but not in subjects with diabetes mellitus type 2. Eur. J. Clin. Investig..

[49] Türk Y., Theel W., Kasteleyn M.J., Franssen F.M.E., Hiemstra P.S., Rudolphus A., Taube C., Braunstahl G. (2017). High intensity training in obesity: A Meta-analysis. Obes. Sci. Pract..

[50] Hansen D., Niebauer J., Cornelissen V., Barna O., Neunhäuserer D., Stettler C., Tonoli C., Greco E., Fagard R., Coninx K. (2018). Exercise Prescription in Patients with Different Combinations of Cardiovascular Disease Risk Factors: A Consensus Statement from the EXPERT Working Group. Sports Med..

[51] Battista F., Belligoli A., Neunhaeuserer D., Gasperetti A., Bettini S., Compagnin C., Marchese R., Quinto G., Bergamin M., Vettor R. (2021). Metabolic Response to Submaximal and Maximal Exercise in People with Severe Obesity, Prediabetes, and Diabetes. Obes. Facts.

[52] Vecchiato M., Neunhaeuserer D., Quinto G., Bettini S., Gasperetti A., Battista F., Vianello A., Vettor R., Busetto L., Ermolao A. (2021). Cardiopulmonary exercise testing in patients with moderate-severe obesity: A clinical evaluation tool for OSA?. Sleep Breath..

